# Localizing WHO SMART guidelines-Digital Adaptation Kits (DAKs) for country impact: implementation insights from Cameroon, Ethiopia, Ghana, and Zambia

**DOI:** 10.1093/oodh/oqag013

**Published:** 2026-06-07

**Authors:** Rosemary K Muliokela, Miriam Nkangu, Kuwani Banda, Abdulaziz M Hussen, Selamsew T Tsigie, Kelvin Sikwibele, Jessica Asante, Angel Mwiche, Emmanuel Batoum, Ngoi Kalamatila, Zach N Ebongo, Azmach Hadush, Binyam Tilahun, Chris Guure, Tigest Tamrat

**Affiliations:** UNDP/UNFPA/UNICEF/WHO/World Bank Special Programme of Research, Development and Research Training in Human Reproduction (HRP), Department of Sexual, Reproductive, Maternal, Child, Adolescent Health and Ageing, World Health Organization, Avenue Appia 20, 1211 Geneva 27, Switzerland; Digital & Global Health Lab, Bruyere Health Research Institute, 43 Bruyère Street, Ottawa, ON K1N 5C8, Canada; Institute for Health Measurement (IHM) Southern Africa, Quantum Office Park, Platinum Road, Ibex Hill, Lusaka, Zambia; Digital Health and Implementation Science, University of Gondar, Addis Ababa, Ethiopia; Department of Global Health and Bioethics, Julius Centre for Health Sciences and Primary Care, University Medical Centre Utrecht, Utrecht University, Heidelberglaan 1003584 CX Utrecht, Netherlands; Digital Health and Implementation Science, University of Gondar, Addis Ababa, Ethiopia; Institute for Health Measurement (IHM) Southern Africa, Quantum Office Park, Platinum Road, Ibex Hill, Lusaka, Zambia; Department of Biostatistics, School of Public Health, University of Ghana, Legon-Accra, Ghana; Department of Public Health, Ministry of Health, Haile Selassie Avenue, Lusaka, Zambia; Department of Information Technology, Ministry of Public Health, N° 8, Rue 3038, Quartier du Lac, Yaounde, Cameroon; SMART Zambia Institute, Office of the President, E-Government Division, Corner of Independence Avenue and Nationalist Road, Lusaka, Zambia; Department of Family Health, Ministry of Public Health, N° 8, Rue 3038, Quartier du Lac, Yaounde, Cameroon; Health Systems Cluster, World Health Organization Country office, Plot 4609, UN Annex Building, Corner of Andrew Mwenya Road and Beit Road, Rhodes Park, Lusaka, Zambia; Digital Health and Implementation Science, University of Gondar, Addis Ababa, Ethiopia; Department of Health Informatics, Institute of Public Health, College of Medicine and Health Science, University of Gonder, Gonder, Ethiopia; Department of Biostatistics, School of Public Health, University of Ghana, Legon-Accra, Ghana; UNDP/UNFPA/UNICEF/WHO/World Bank Special Programme of Research, Development and Research Training in Human Reproduction (HRP), Department of Sexual, Reproductive, Maternal, Child, Adolescent Health and Ageing, World Health Organization, Avenue Appia 20, 1211 Geneva 27, Switzerland

**Keywords:** electronic health records, Digital Adaptation Kits, standards, guidelines, localization, digital health governance, clinical decision support, sustainability, digital health implementation, sexual reproductive health, family planning, antenatal care

## Abstract

Global investments in digital health are accelerating progress toward universal health coverage, yet low- and middle-income countries face persistent challenges: fragmented data and limited interoperability inhibit systematic transformation. The World Health Organization’s SMART (Standards-based, Machine-readable, Adaptive, Requirements-based, and Testable) guidelines and Digital Adaptation Kits (DAKs) offer a standardized, evidence-based framework that enables countries to localize digital content and reinforce the uptake of standards at the point of care. Lessons from early adopters, including Cameroon, Ethiopia, Ghana, and Zambia, demonstrate that structured DAK localization strengthens alignment with national digital strategies and the potential to harmonize information systems. Effective adoption is driven by early user engagement, strong government leadership, and multisectorial collaboration. Mainstreaming the SMART Guidelines approach highlights the importance of robust governance and contextual adaptation in sustaining and scaling digital solutions that align with global standards.

## Introduction

As countries navigate the complexities of the rapidly evolving global health landscape, governments are increasing investments in digital health to build stronger, more efficient, and resilient health systems that will not only confront emerging challenges but also elevate the level of care to achieve universal health coverage (UHC) (World Health Organization 2018, World Health Organization 2021a, Transform Health 2023, World Bank 2023, Forslund et al. 2024).

However, achieving digital health transformation remains a significant challenge. Many health systems, especially in low and middle-income countries (LMICs), continue to struggle with fragmented data and systems, limited interoperability, workforce capacity gaps, and inadequate digital governance (World Health Organization 2021a, Forslund et al. 2024, Kozlakidis et al. 2024, Adegoke et al. 2025, Alotaibi et al. 2025). Moreover, the integration of health and data recommendations into digital systems has also been unsystematic and error-prone (World Health Organization n.d., Mehl et al. 2021, Tamrat et al. 2022). This results in missed opportunities to fully harness the potential of digital tools to advance UHC (Forslund et al. 2024).

**Figure 1:**
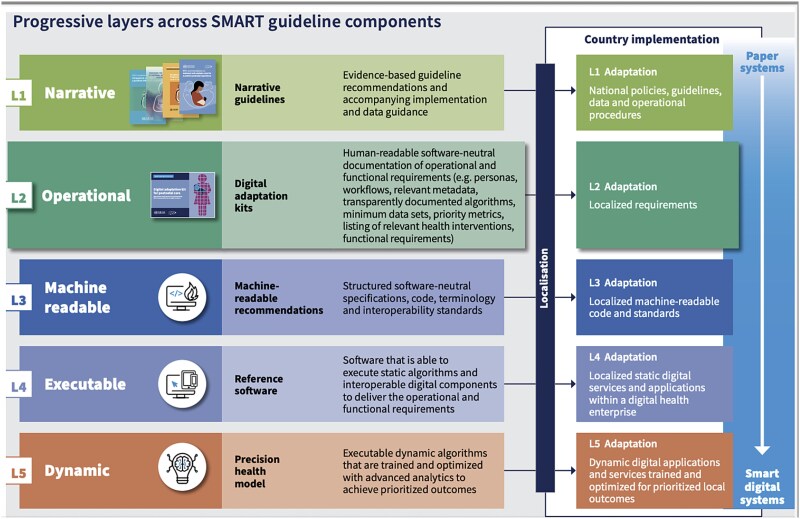
DAKs within the framework of SMART guidelines.

To address these gaps and provide a framework for integration of standards and evidence-based practices during the process of digitalization, and reinforce their uptake at the point-of-care, the World Health Organization (WHO) established the SMART—Standards-based, Machine-readable, Adaptive, Requirements-based, and Testable—guidelines framework in 2021 (Fig. 1) (Mehl et al. 2021, Tamrat et al. 2022). A key foundational component of this framework is the Digital Adaptation Kits (DAKs), which translate normative WHO guidance into operational software-neutral packages. DAKs are packaged as one main operational document, typically in PDF format, accompanied by four web-based annexes in Excel format that include: (World Health Organization 2021a) core data elements and a data dictionary defining key data items and their standardized descriptions; (Transform Health 2023) decision-support tables providing logic and algorithms for decision making at the point of care; (Forslund et al. 2024) program indicator definitions for monitoring and data aggregation; (World Health Organization, 2018) functional and non-functional requirements describing what the digital must do and how it should perform(See Appendices 1 and 2) (Mehl et al. 2021, Tamrat et al. 2022). DAKs are software-agnostic packages that enable countries to localize and adapt to national health programs and digital landscapes, including settings with existing digital systems as well as those that are planning or transitioning from paper or legacy systems (World Health Organization 2024a, Muliokela et al. 2025). This approach ensures that digital content remains quality-assured, standardized, and interoperable across a wide range of platforms (Mehl et al. 2021, Muliokela et al. 2025, Tamrat et al. 2025). To date, DAKs have been developed for antenatal care (ANC) (World Health Organization 2021b), family planning (FP) (World Health Organization 2021c), HIV/AIDS (World Health Organization 2022), immunization (World Health Organization 2024b), tuberculosis (TB) (World Health Organization 2024c), child health in humanitarian emergencies (World Health Organization 2024d), and postnatal care (World Health Organization 2025a). Additional DAKs in development include intrapartum care, community health, and other health areas to support the entire spectrum of digitalizing primary health care (PHC).

Drawing from country experience gained through targeted technical support to member states, as well as implementation research—Guideline Uptake in Digital Ecosystems (GUIDE) study (Muliokela et al. 2025, Tamrat et al. 2025) and others, we are developing and refining methodologies for the localization and uptake of DAKs, as a pathway to standardize and promote quality of care through digital investments. In this article, we highlight these processes and share practical localization and implementation insights from selected early adopter countries (Cameroon, Ethiopia, Ghana, and Zambia) underpinning this emerging, standards-enabled approach to digitalization.

## DAK localization methodologies and emerging considerations

**Figure 2:**
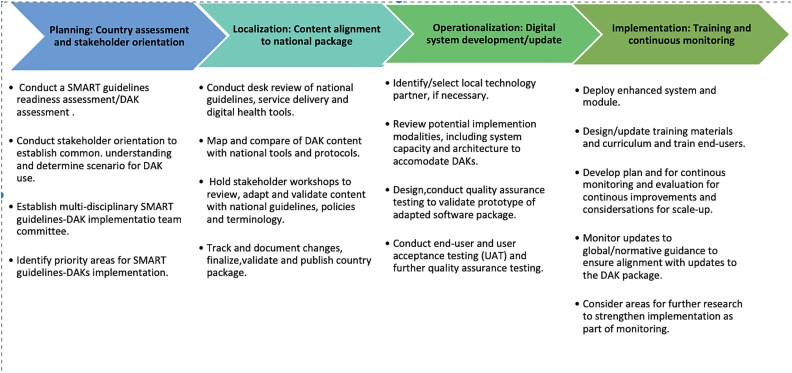
Overview of DAK country implementation processes and steps.

The structured approach and methodologies for DAK localization were shaped by insights from Zambia, where the initial methodology was developed, and further refined and standardized through implementation research in Ethiopia and Ghana. Cameroon later adopted the methodology independently to upgrade its system. Building on these experiences, a multi-step, replicable methodology for DAK localization was defined, which included: (World Health Organization 2021a) country assessment (Transform Health 2023); localization; (3)Operationalization-system development; and (World Health Organization 2018) implementation- training and continuous monitoring (Fig. 2) (World Health Organization 2025b). Across these steps, countries systemarically reviewed the generic WHO DAK and aligned it with national protocols, guidelines, and data use standards to ensure contextual relevance and consistency within their respective digital systems (Muliokela et al. 2025, Tamrat et al. 2025, Nkangu et al. 2025).

### Country assessment and implementation coordination

The initial step is to introduce the DAKs through a country assessment, which also examines national guidelines and digital landscape to establish a common understanding of the localization approach (Muliokela et al. 2025, World Health Organization 2025b). When introducing the DAK concept and SMART Guidelines in general, stakeholders may expect a digital tool or application for end-users, such as health workers (Muliokela et al. 2025). As such, it will be important to clarify from the outset that DAKs are content requirements (Muliokela et al. 2025). A key output of this introductory step is the establishment of a multi-stakeholder steering committee to oversee localization (Muliokela et al. 2022, Muliokela et al. 2025, World Health Organization 2025b, Nkangu et al. 2025, Nkangu et al. 2024). This multi-stakeholder committee typically includes clinicians, digital system developers, health information system (HIS) and monitoring and evaluation (M&E) experts, health domain experts, program and digital health leads. Clinicians and program leads ensure that workflows, decision-support logic, and data requirements align with national protocols and frontline realities. Developers and system architects guide the localization process to ensure alignment and compatibility with the target system architecture and also translate the DAK content into software components. HIS and M&E experts validate data elements, indicators, and reporting requirements to ensure alignment with national Health Management Information System (HMIS) standards. Program and digital leads from government provide governance oversight, ensure alignment with national strategies, and support institutional ownership and facilitate implementation, including training, deployment, and roll-out of the DAK-enabled system. Together, these stakeholders conduct iterative reviews, validation workshops, and mapping exercises to contextualize the DAK to country needs while ensuring accuracy, feasibility, and alignment with existing systems.

### Localization, adaptation, and validation

Once priority areas for localization, including the relevant DAK domain, have been identified through stakeholder workshops, the generic DAK content is subsequently reviewed, adapted, and validated against national guidance (Muliokela et al. 2025, Tamrat et al. 2025, World Health Organization 2025b, Nkangu et al. 2025, Nkangu et al. 2024). This process begins with pre-mapping, which entails examining generic DAK content (data dictionary/decision support logic) against the equivalent national protocol content to determine the degree of alignment or divergence (Muliokela et al. 2025, Tamrat et al. 2025, World Health Organization 2025b). The key outputs of the pre-mapping process include adaptations that are categorized as follows: (i) adoptions(content taken exactly as presented in the WHO DAK as it aligns with national protocols); (ii) modifications(content aligned in concept but require rewording/adjustment to fit national standards; (iii) removals (content from the WHO DAK that doesn’t reflect national practice, and thus isn’t incorporated, and; (iv) additions (new elements introduced from national protocols or digital systems that were absent from the DAK. For example, if localizing the ANC DAK, the data dictionary element ‘Pelvic exam’ under the physical examination section would be compared with and ‘mapped’ to the corresponding national protocol term ‘Pelvic assessment’ (World Health Organization 2021b). Where both data elements describe the same clinical concept but differ in terminology or structure, this would be classified as a ‘modification’. All proposed adaptations are documented and tracked in a country adaptation log (Appendix 3). This documentation is essential for both country-level implementation and the broader global DAK development and normative guidance processes, ensuring that updates and refinements reflect frontline realities (Muliokela et al. 2025, Tamrat et al. 2025, World Health Organization 2025b).

The next step is the validation of the draft package, which typically involves the steering committee, government program, and digital leads, and other relevant health authorities reviewing the adaptation log, including all adoptions, modifications, additions, and removals (Muliokela et al. 2025, Tamrat et al. 2025, World Health Organization 2025b). These stakeholders then formally approve the adapted components to ensure consistency with national protocols and digital strategies. Any additional revisions are documented as part of the iterative refinement process (Muliokela et al. 2025, Tamrat et al. 2025, World Health Organization 2025b).

### Digital system content updates, training, testing, and deployment

Once the localized package has been validated, it is shared with system developers to implement or integrate content within the designated digital platform, following standard software development life cycle processes (Muliokela et al. 2025, World Health Organization 2025b, Nkangu et al. 2025). For many countries, adopting the DAK approach and transitioning from data-centric systems to patient-centered decision support systems represents a new and often challenging shift (World Health Organization 2025b). It will be crucial to conduct multiple rounds of internal quality assurance testing with system developers and clinicians before proceeding to actual end-user testing (World Health Organization 2025b). This iterative validation ensures that inputs and outputs are displayed accurately and function as intended (World Health Organization 2025b). Once this is completed, the DAK enhanced system or module is ready for deployment. To facilitate effective system adoption during roll-out, it will be important to ensure that health workers and clinicians receive training on the updated system during the initial roll-out and continue to be supported through refresher trainings and ongoing mentorship throughout implementation. Such sustained support is critical to enabling consistent and effective use of the system in routine practice.

### Implementation, continuous support, and monitoring

The final step, an ongoing process, involves monitoring the system and aligning updates to ensure that the DAK package embedded within the system remains consistent with evolving guidance and clinical protocols. These updates help maintain accuracy, relevance, and compliance with national and global standards over time. All the processes described above are detailed in the DAK implementation facilitation guide, a forthcoming WHO technical resource to support countries in applying this approach for their digitalization journeys (World Health Organization 2025b).

## Country insights and lessons learned

Early adopter countries of the SMART Guidelines-DAK approach are demonstrating, through a combination of stakeholder-reported implementation observations and early research insights—how DAKs can be incorporated into real-world applications, bridging the gap between evidence-based health guidelines and routine health service delivery. The countries represented in this paper are among the first to apply this approach to enhance existing digital systems through structured implementation and research efforts.

For Zambia, insights presented here primarily reflect early implementation observations and stakeholder perceptions, including those of clinicians, program and digital leads, and software developers—assessed on the basis of implementation reports and experience adapting the FP DAKs. Zambia is among the first countries to implement this structured approach, drawing on experience from implementing FP DAKs as part of the broader United Nations interagency initiative *2gether4SRHR* (World Health Organization Regional Office for Africa, 2017), an initiative that included leveraging digital tools to strengthen sexual and reproductive health and rights as an approach (Muliokela et al. 2025, World Health Organization 2021c). These perspectives were captured through country workshops and iterative consultations through the localization process.

Insights from Ethiopia and Ghana stem from the WHO GUIDE study (Tamrat et al. 2025). The GUIDE study is one of the first research efforts designed to evaluate the impact of DAKs on service delivery outcomes (Muliokela et al. 2025, Tamrat et al. 2025). This two-phase implementation research first determined the requirements for localizing DAKs for national digital systems: Bahmni in Ethiopia and e-tracker in Ghana. The second phase focuses on evaluating the impact of the DAK upgraded systems on service delivery outcomes (Tamrat et al. 2025).

The initial research findings documented here draw on implementation reports prepared by the research teams at the Universities of Gondar and Ghana. These reports drew from stakeholder inputs, including software developers and frontline providers, gathered through workshops and iterative consultations. Together, these perspectives provided early insights into the DAK validation and user-testing processes in Ghana and the DAK system integration and deployment processes in Ethiopia.

Although Cameroon was not part of either the GUIDE research study or the WHO country implementation initiative, established DAK methodologies and tools were applied in close consultation with WHO to design and upgrade the BornFyne Prenatal Management System (PNMS) using the ANC DAK (World Health Organization 2021b, Muliokela et al. 2022). Insights for this paper were also derived from stakeholder workshops and from the BornFyne-PNMS study team’s documented implementation processes, including a pre-post assessment during the training (Nkangu et al. 2025, Nkangu et al. 2024). This system is currently being piloted in Cameroon (Nkangu et al. 2025, Nkangu et al. 2024).

The primary observation window for all countries was 2022–2025. No new individual-level data were collected or analyzed; all findings presented here are based on stakeholder-reported implementation observations and early research insights, with formal outcome evaluations ongoing where applicable.

The following sections highlight each country’s distinct yet complementary experiences applying the DAK approach within digital systems of varying maturity (Table 1). These examples underscore the importance of contextual adaptation, robust governance structures, and early engagement of policymakers and implementers to ensure alignment with national digital health architectures. Collectively, they offer practical insights to inform global replication and scale-up of the DAK approach.

**Table 1:** Summary of country localization insights.

**Country**	**Platform**	**DAK domain(s)**	**Implementation stage**	**Key adaptation highlights**	**Key challenges**	**Mitigation measures**
**Zambia**	SmartCare (National EHR; mature digital system)	FP	Localization for scale	HMIS alignment with FP DAK Adoption into national digital health policy and EHR requirements	Limited existing FP content requirements Large volume of FP DAK data elements prolonged localization	Early stakeholder engagement Existing system walk throughs Iterative content validation
**Cameroon**	BornFyne PNMS (mobile application; early stage digital maturity)	ANC	Integration and deployment through a pilot	Alignment with health facility registers Addition of malaria-related data elements	Lack of comprehensive ANC normative guidance No prior ANC digital system to reference Dependence on facility registers created interpretation gaps	Cross-functional content validation team (MoH, clinicians, developers) Localization process informed updates to national ANC guidance
**Ethiopia**	Bahmni EMR (early stage digital maturity)	FP, ANC	Integration and deployment through research	Mapping of DAK data dictionary and workflows Reusable integration components for future scale	Time-intensive mapping and translation of components Infrastructure issues (power)	Advance planning with skilled technical resources Structured training + addressing infrastructure constraints during rollout
**Ghana**	DHIS2 eTracker (National Platform)	FP, ANC, HIV	Integration and deployment through research	Tailored decision-support prompts Workflow harmonization with GHS protocols	Limited familiarity with digital decision-support systems Large volume of DAK data elements risked duplication	Hands-on, scenario-based training Iterative user testing and refinement cycles Content prioritization to reduce workflow burden

### Localizing DAKs to optimize national electronic health records for sustainability and scale: Zambia

Zambia offers key insights into localizing DAKs for electronic health records (EHRs) within an evolving digital ecosystem (The Global Fund 2025). Zambia’s Digital Health Strategy 2022–26 underscores the Government’s strong commitment to standardizing evidence-based digital content and prioritizing decision-support integration in SmartCare, the national EHR (Digital Health Strategy Zambia 2022). Pre-mapping DAK content with existing workflows helped identify areas requiring adaptation prior to stakeholder workshops. Early engagement proved essential, as the localization process revealed additional gaps and highlighted the need for continuous, iterative alignment among technical teams, program managers, and frontline providers to ensure that DAK content accurately reflects the national service delivery context (Muliokela et al. 2025).

A key highlight of Zambia’s approach was harmonization of the localized FP DAK with the national HMIS (Republic of Zambia, Ministry of Health 2023, Muliokela et al. 2025). As reported by program and HIS experts, aligning data standards, indicators, and reporting structures ensured point-of-care decision-support logic remained consistent with national reporting and monitoring requirements. Stakeholders perceived that this alignment strengthened the linkage between clinical workflows and routine reporting and would support more effective near-real-time feedback loops between service-delivery data and program decisions (Kiberu et al. 2014, Kessy et al. 2024).

Zambia’s localization efforts led to the adoption of the DAK approach as the standard for defining EHR requirements documentation. Additionally, the approach was leveraged as a strategy to guide sexual reproductive health (SRH) digitalization, evidenced by its integration into national FP protocols and guidance (Republic of Zambia, Ministry of Health n.d.). By embedding the DAK approach within national health policies, the Ministry of Health ensured that DAK initiatives were strategically aligned with broader health priorities—an essential step toward sustainable digital transformation. Key challenges included the inadequate availability of existing content requirements for FP and the large volume of data elements contained within the FP DAK, both of which contributed to a more time-intensive localization process (Muliokela et al. 2025). Localization teams relied on the existing system content and the generic DAK as a starting point and complemented this with multiple iterative stakeholder consultations to verify and validate content against actual clinical practice.

### Leveraging the DAK approach to bridge gaps in normative guidance for adaptation of digital solutions: Cameroon

Cameroon’s National Digital Health Strategy (2020–24), which is currently under revision, underscores the importance of standardized clinical guidelines to improve the quality of care, strengthen provider adherence, and build capacity (Republic of Cameroon, Ministry of Public Health, Cameroon 2024). Integrating guideline-based digital tools is viewed as essential for enhancing interoperability of systems, advancing nationwide digitalization, and accelerating progress toward UHC (Nkangu et al. 2025, Republic of Cameroon, Ministry of Public Health, Cameroon 2024). The Cameroon experience demonstrates the practical application of the ANC DAK through its integration into the BornFyne PNMS (World Health Organization 2021b, Nkangu et al. 2025).

This process involved content validation with a small team of clinicians, developers, and digital and program leads from the Ministry of Public Health, who reviewed the generic DAK against health facility registers and subsequently approved the country-adapted DAK package. The approved DAK package was then integrated into BornFyne-PNMS ANC module (Nkangu et al. 2025). Key adaptations included aligning DAK elements with facility registers and adding malaria-related data elements (Nkangu et al. 2025). Pilot trainings using the DAK-enhanced BornFyne platform demonstrated improved provider adherence to protocols, with perceived quality-of-care scores reflecting a better understanding of comprehensive, patient-centered care aligned with global best practices (Nkangu et al. 2024, Chelwa et al. 2025, World Health Organization 2016). The process also raised stakeholder awareness of standardized ANC and promoted collaboration across sectors (Nkangu et al. 2025, Nkangu et al. 2024).

A major challenge was the absence of comprehensive normative guidance for ANC, which became evident during the localization process. Additionally, because no prior digital ANC system existed before the pilot, contextualization of the DAK relied primarily on facility registers and inputs from frontline stakeholders. This limited the contextualization of the DAK to only facility registers and frontline stakeholder consultations, often prolonging the localization process and limiting the ability to comprehensively capture all required elements (Nkangu et al. 2025, Nkangu et al. 2024). Nevertheless, the process catalyzed national-level discussions aimed at updating and harmonizing ANC normative guidelines and, recently, the updated national norms and standards for SRH now provide guiding framework for adapting comprehensive clinical guidelines within the national health system. Similar to the experience in Zambia, the generic ANC DAK served as a critical starting point for organizing localization documentation and guiding national adaptation efforts.

### Early insights from DAK enhanced e-tracker system user testing: Ghana

Ghana’s experience under the GUIDE study highlights the critical role of early and structured engagement with users and stakeholders in localizing content and customizing DAK software packages for ANC, FP, and HIV for integration into the national eTracker platform (Muliokela et al. 2025, Tamrat et al. 2025). This approach ensured that adapted packages reflected frontline realities, aligned with clinical workflows, and fostered ownership—a critical factor for effective system adoption (Akwaowo et al. 2022). Key adaptations included tailoring decision-support prompts, counseling messages, and referral workflows to align with Ghana Health Service (GHS) protocols and service-delivery practices.

Following localization and validation of the DAK content, DAK-enhanced modules were deployed for testing in eight pilot facilities across Ghana’s Eastern and Upper East regions. System rollout began with a structured user acceptance testing workshop that brought together frontline healthcare workers, GHS representatives, and other stakeholders who had participated in earlier adaptation and ingestion processes. This diverse mix of participants helped align expectations, clarify clinical workflows, and resolve queries in real time, while fostering joint ownership of the adapted DAK content and its integration into the eTracker platform.

The Ghana implementation also surfaced key challenges. Limited familiarity among some users with digital decision-support systems required additional orientation. Moreover, integrating the large volume of DAK data elements into an already operational national platform demanded careful content prioritization and harmonization to prevent duplication and workflow burden.

Hands-on training using real-world scenarios, particularly those reflecting the decision support component, a new feature of the upgraded system, and iterative user testing proved essential for building confidence and competency among health workers in using the DAK-enabled system.

### DAK system integration and early deployment lessons: Ethiopia

As part of the GUIDE study, Ethiopia successfully localized and integrated the FP and ANC DAK packages into the Bahmni Electronic Medical Record (EMR) system (Tamrat et al. 2025). This experience demonstrated that integrating DAKs into existing digital health platforms, each with distinct languages, data models, and architectures, requires a structured, systematic, and future-proof approach. A critical first step involves mapping and translating all fields and attributes in the DAK data dictionary and decision-support components. One of the earliest challenges was the time-intensive process of mapping and translating all fields and attributes in the DAK data dictionary and decision-support components, reinforcing the need for adequate planning and allocation of technical resources during the integration phase. The Ethiopia experience further demonstrated that software components should be designed with versatility in mind, ensuring separation between content, data, and configurations and the core software elements. This approach maximizes adaptability and scalability, particularly when updating DAK-enabled modules within a digital system. While automation can reduce manual workload and errors, it must be paired with rigorous technical and clinical validation, including iterative testing with clinicians and health domain experts, to ensure accuracy and alignment with DAK specifications.

Early assessments revealed that health workers increasingly valued the system for service delivery and decision-making, highlighting the importance of structured training at deployment, mentorship during early stages, and addressing infrastructure challenges such as power reliability and system performance to drive adoption and effective utilization.

### Mainstreaming SMART guideline DAKs adoption: government and private sector perspectives

The Government of Zambia has played a pivotal role in advancing digital health transformation by embedding global standards into national information technology and communication (ICT) frameworks and health-sector platforms (SMART Zambia Institute 2023). Through the SMART Zambia Institute (SZI), DAK-aligned principles have been incorporated into key ICT governance instruments such as the Public Service ICT Standards: Systems Acquisition Guideline (SMART Zambia Institute 2023) and the eGovernment Interoperability Standard (Akwaowo et al. 2022). In the health sector, SmartCare Pro, Zambia’s national EHR, is being upgraded using the DAKs (SMART Zambia Institute 2022).

While Zambia has made significant national progress in integrating DAKs into its national health system, other DAK early adopter countries are also advancing. Cameroon is renewing its national digital health strategy, and insights from the BornFyne pilot research project have strengthened government advocacy for mainstreaming the SMART Guidelines approach as part of broader digital transformation efforts (Republic of Zambia, Ministry of Health n.d.). Similarly, Ghana and Ethiopia continue to strengthen alignment with standardized, interoperable digital health frameworks through ongoing research and plan to leverage post-study learnings to scale up and mainstream this approach within their broader digital ecosystems.

Zambia’s experience demonstrates that early adoption and integration of standards establishes a strong foundation for sustainable national DAK implementation and adoption. This approach enables mainstreaming across multiple health domains while leveraging lessons learned for broader ecosystem digital transformation.

## Discussion

Experiences from Zambia, Cameroon, Ghana, and Ethiopia illustrate that the SMART Guidelines–DAK approach can be effectively applied across diverse digital health ecosystems, despite varying levels of digital maturity. Across all four settings, DAKs provided a structured mechanism for translating WHO guidance into digital systems, strengthening clinical workflows, and aligning point-of-care decision-support with national reporting requirements (Tamrat et al. 2022). Zambia’s national-scale implementation highlighted the importance of early structured pre-mapping, strong alignment with national policies, and harmonization with the HMIS are critical to ensure scalability and long-term sustainability, consistent with findings from the broader literature (Labrique et al. 2018, Labrique et al. 2020).

Cameroon showed how DAK-enabled digitalization can strengthen adherence to care standards. This is consistent with evidence from Nepal, where an antenatal care digital decision support tool designed using the DAKs was associated with improved counseling, as clinicians benefited from decision support prompts (Karmacharya et al. 2025). Similar experiences have been reported elsewhere, where integrating decision-support algorithms into digital platforms (Michaels 2023). Additionally, the experience in Cameroon revealed gaps in normative guidance, underscoring the need for continuous review and updating of national protocols and aligning with digital systems (Michaels 2023). Ghana’s experience emphasized the importance of user-centered processes. Hands-on training and iterative testing were central to improving usability and fostering provider ownership, findings consistent with evidence from Ghana and other similar settings highlighting the role of user engagement in digital system uptake and adoption (Pellé et al. 2020, Achampong 2022, Muliokela et al. 2025, Tamrat et al. 2025). Ethiopia demonstrated that successful integration into an existing EMR requires systematic data mapping, modular configuration for reusability, and rigorous clinical and technical validation. Ghana’s experience with integration underscored the need for careful planning and prioritization of content for integration to avoid workflow disruption of existing and operational digital platforms.

Several shared challenges also emerged across countries. The large volume of DAK content, particularly for FP DAK, contributed to prolonged localization timelines (Muliokela et al. 2025). However, as highlighted by earlier implementation learnings, conducting preparatory activities prior to pre-mapping, including structured guideline extraction to identify key areas for localization, can help mitigate these delays (Muliokela et al. 2025, World Health Organization 2025b). Moreover, exploring artificial intelligence (AI) to accelerate the localization processes may be a further consideration (World Health Organization 2025b). Limited existing system documentation, as observed in Cameroon and Zambia, further slowed the contextualization process and increased reliance on stakeholder interviews or system walkthroughs. Nevertheless, the less, the DAKs proved to be a useful generic starting point for transparent and systematic consultations to fill in the identified ‘knowledge gaps.’ (Muliokela et al. 2025, World Health Organization 2025b).

Another cross-cutting challenge was striking the right balance between providing adequate decision support and maintaining streamlined system workflows. Ensuring that additional decision-support functionalities enhance rather than disrupt the ‘natural’ or existing user workflow remains a delicate design consideration. Infrastructure constraints, particularly intermittent power supply and system-performance issues, posed a significant challenge for Ethiopia, limiting the usability of the DAK-enhanced Bahmni system during the early stages of rollout. These findings are consistent with broader evidence from the region: studies from Ghana and across sub-Saharan Africa similarly identify unreliable electricity, hardware limitations, and system slowdowns as major barriers to effective EHR adoption and sustainability (Mensah et al. 2024, Mugauri et al. 2025). These findings reinforce the need for digitalization initiatives to be paired with investments in foundational infrastructure and enabling environments—including backup energy solutions—to ensure consistent system use and long-term impact.

These findings are constrained by the early stage of implementation, with insights drawn primarily from stakeholder experiences. Ongoing evaluations under the WHO GUIDE study are expected to provide more rigorous evidence on how DAK-enabled systems impact clinical workflows, service quality, and guideline adherence at the point of care. The study will also help refine localization and integration processes and methodologies, providing critical insights to strengthen future implementation strategies.

Across all settings, several practices emerged as important for replication: sustained engagement of ministries, implementers, and frontline users; embedding standards within national ICT, and governance and program frameworks; targeted capacity building through training and mentorship; and phased, iterative deployment to refine systems before scale-up. Collectively, early adopter experiences show that, when grounded in strong governance and contextual adaptation, the SMART Guidelines–DAK approach offers a scalable and interoperable pathway for advancing national digital health transformation.

## Conclusion

By emphasizing the integration of WHO SMART Guidelines and DAKs into Cameroon, Ethiopia, Ghana, and Zambia’s health ecosystems, this paper provides valuable lessons for countries looking to leverage this approach to build and enhance patient-centered point-of-care systems at PHC level. Emerging common themes include the necessity of aligning digital solutions with existing national policies, standards, and data frameworks; prioritizing local capacity-building efforts to foster ownership and sustainability; and establishing comprehensive governance structures capable of supporting scalable implementations. The WHO SMART Guidelines and DAKs represent an opportunity for enhancing global health through digitalization, offering tools and strategies that have the potential to promote sustainability, scalability, and equity to build resilient digital systems.

## Supplementary Material

SMG_DAKs_Oxford_list_of_Appendices_May_28_2026_OXFORD_oqag013

## Data Availability

No new data were generated or analyzed in support of this article.
