# Supplementary material for: Localizing WHO SMART guidelines-Digital Adaptation Kits (DAKs) for country impact: implementation insights from Cameroon, Ethiopia, Ghana, and Zambia
Source: Oxf Open Digit Health. 2026 Jun 7;4:oqag013. doi: 10.1093/oodh/oqag013 (PMC13370239; doi:10.1093/oodh/oqag013)

Appendix 1

**Table 1. Components of a digital adaptation kit**

|  | **Component** | **Description** | **Outputs/ artifacts** | **Adaptation needed** |
| --- | --- | --- | --- | --- |
| 1 | Health interventions and recommendations | Overview of the health interventions and WHO recommendations included in the digital adaptation kit (DAK). DAKs are meant to be a repackaging and integration of WHO guidelines and guidance documents in a particular health domain. The list of health interventions is drawn from the universal health coverage menu of interventions compiled by WHO *(22)*. | **List of related health interventions** based on WHO’s universal health coverage essential interventions.    **List of related WHO recommendations** based on guidelines and guidance documents.  **List of related digital health interventions** based on the *Classification of digital interventions, services and applications in health: a shared language to describe the uses of digital technology for health* *(17).* | Contextualization to reflect current or planned national policies and guidelines. |
| 2 | Generic personas | Depiction of the end-users and related stakeholders who would be interacting with the digital system or involved in the care pathway. | **Description, competencies and essential interventions** performed by targeted personas. | Greater specification and details on the end-users based on real people (for example, health workers) in a given context.  High-level information to describe the provider of the health service (for example, general background, roles and responsibilities, motivations, challenges and environmental factors). |
| 3 | User scenarios | Narratives that describe how the different personas may interact with the digital system and with each other.  The user scenarios are only illustrative and are intended to give an idea of a typical workflow. | Example **narrative** of how the personas may interact with the system and with each other during a workflow. | Greater specification and details on the real needs of end-users in a given context |
| 4 | Generic business processes and workflows | A business process is a set of related activities or tasks performed together to achieve the objectives of the health programme area, such as registration, counselling, referrals *(1, 2)*.  Workflows are a visual representation of the progression of activities (tasks, decision points, interactions) that are performed within the business process *(1, 2)*. | Overview **matrix** presenting the **key processes.**    **Workflows** for identified business processes with annotations. | Customization of the workflows that can include additional forks, alternative pathways or entirely new workflows. |
| 5 | Core data elements | Data elements are required at different points throughout the workflow.  These data elements are mapped to standards-based classifications and terminologies to ensure that the data dictionary is compatible with other digital systems. | List of **data elements**. | Translation of data labels into the local language and additional data elements created depending on the context. |
| 6 | Decision support logic | Decision support logic and algorithms to support appropriate service delivery in accordance with WHO clinical, public health and data use guidelines. | List of **decisions** that need to be made throughout the clinical encounter | Change of specific thresholds or triggers in a logic (IF/THEN) statements, for example, Body Mass Index (BMI) cut-off, age trigger for youth-friendly services.    Additional decision support logic formulas depending on the context. |
| 7 | Scheduling logic | Scheduling logic to support appropriate reminders for follow-up visits and services in accordance with WHO clinical, public health and data use guidelines. | List of **care plan schedules** in structured format. | Additional scheduling logic depending on the context. |
| 8 | Indicators and performance metrics | Core set of indicators that need to be aggregated for decision-making, performance metrics and subnational and national reporting.  These indicators and metrics are based on data that can feasibly be captured from a routine digital system, rather than from survey-based tools. | **List of indicators table** with numerators and denominators of data elements for calculation, along with appropriate disaggregation. | Changing calculation formulas of indicators.    Adding indicators.    Changing the definition of the primary data elements used to calculate the indicator based on the available data. |

Appendix 2

**Example of WHO FP DAK Data dictionary**


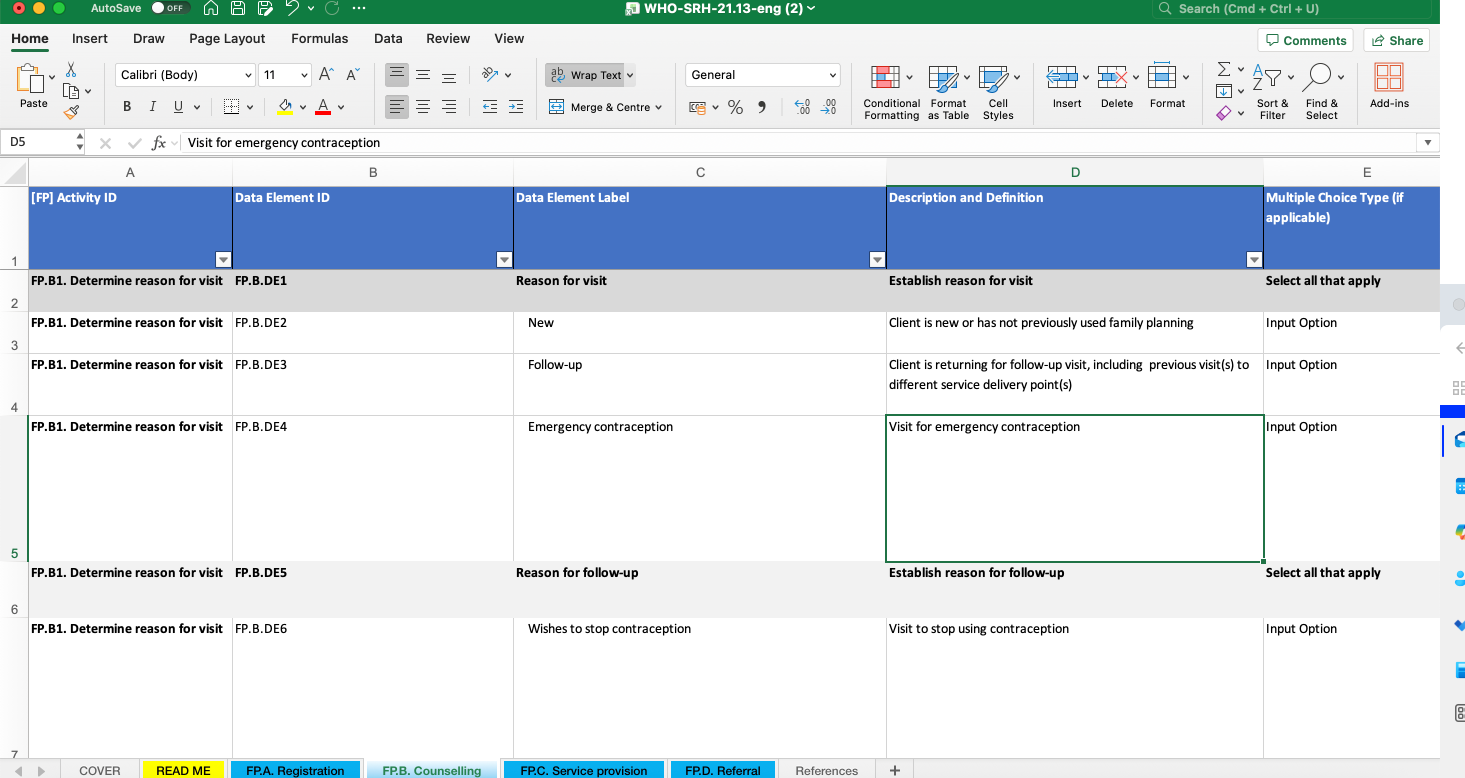


**Example of FP DAK decision support logic tables**


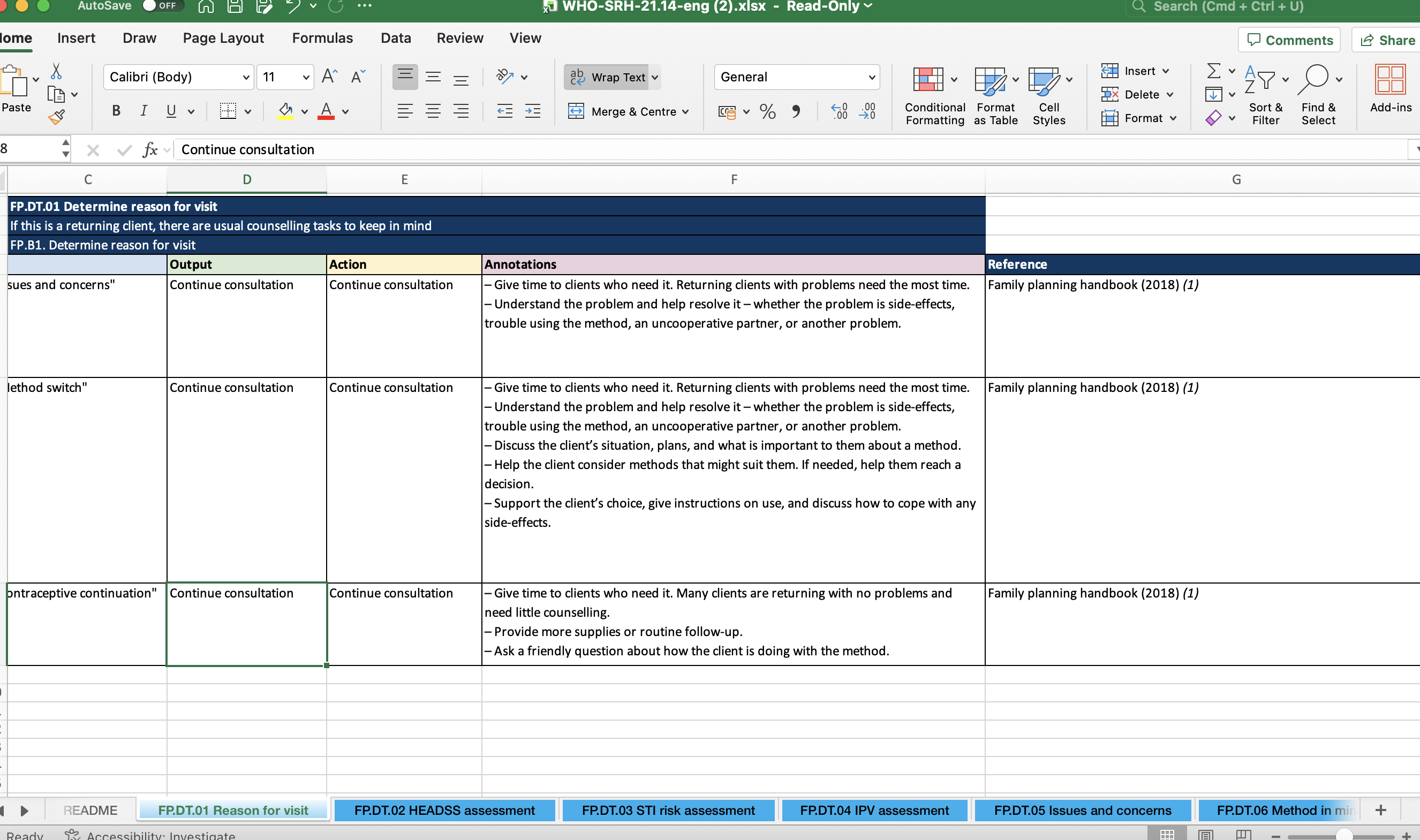


**Appendix 3 Country Adaptation log template**


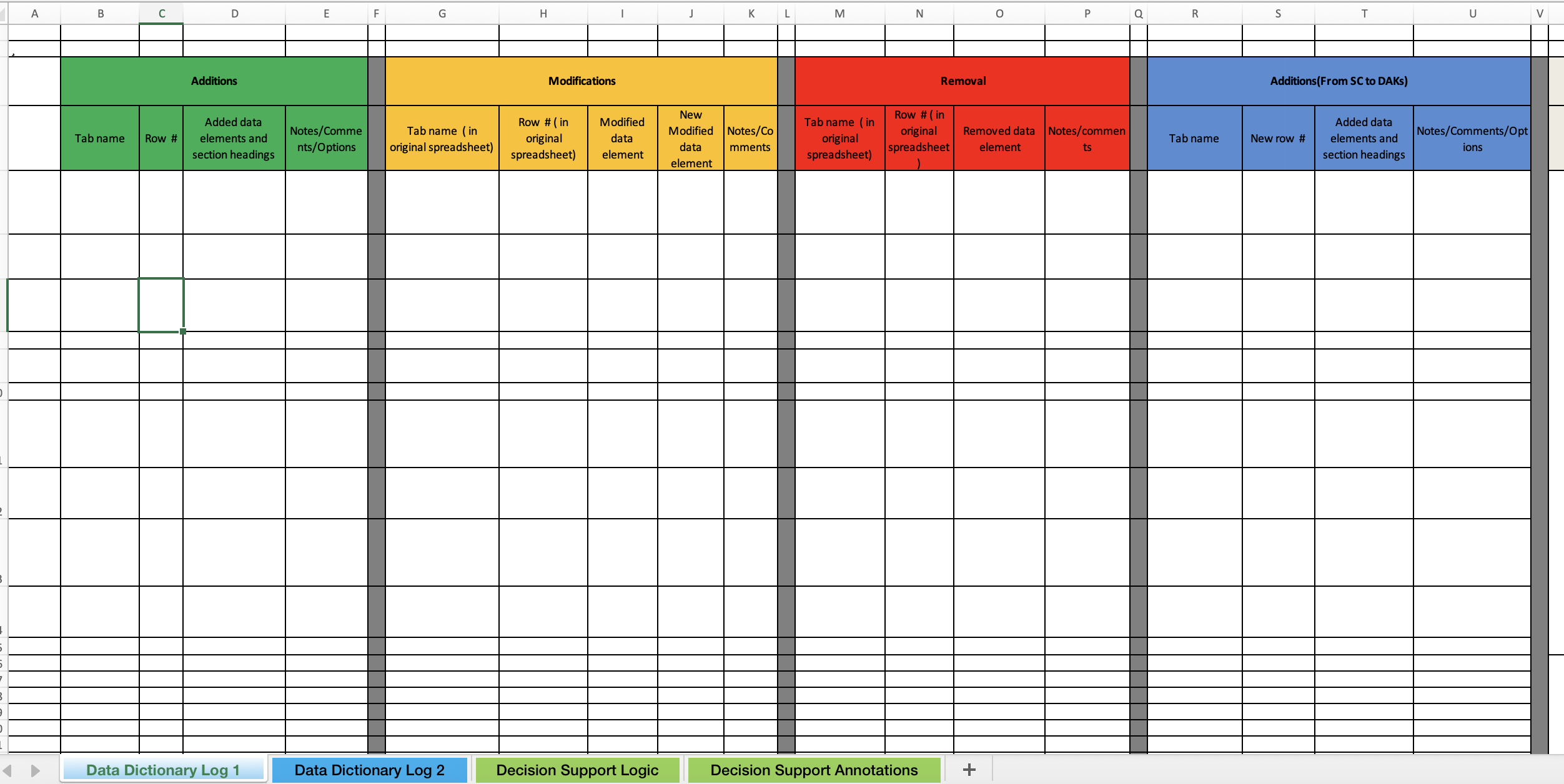

Supplement: SMG_DAKs_Oxford_list_of_Appendices_May_28_2026_OXFORD_oqag013 [file smg_daks_oxford_list_of_appendices_may_28_2026_oxford_oqag013.docx]
